# Mass spectrometry in clinical glycomics: The path from biomarker identification to clinical implementation

**DOI:** 10.1016/j.clinms.2020.08.001

**Published:** 2020-08-27

**Authors:** N. de Haan, M. Wuhrer, L.R. Ruhaak

**Affiliations:** a Center for Proteomics and Metabolomics, Leiden University Medical Center, Leiden, The Netherlands; b Department of Clinical Chemistry and Laboratory Medicine, Leiden University Medical Center, Leiden, The Netherlands

**Keywords:** Mass spectrometry, Glycoprotein, Calibration, Absolute quantitation, Clinical glycomics

## Abstract

Over the past decades, the genome and proteome have been widely explored for biomarker discovery and personalized medicine. However, there is still a large need for improved diagnostics and stratification strategies for a wide range of diseases. Post-translational modification of proteins by glycosylation affects protein structure and function, and glycosylation has been implicated in many prevalent human diseases. Numerous proteins for which the plasma levels are nowadays evaluated in clinical practice are glycoproteins. While the glycosylation of these proteins often changes with disease, their glycosylation status is largely ignored in the clinical setting. Hence, the implementation of glycomic markers in the clinic is still in its infancy. This is for a large part caused by the high complexity of protein glycosylation itself and of the analytical techniques required for their robust quantification. Mass spectrometry-based workflows are particularly suitable for the quantification of glycans and glycoproteins, but still require advances for their transformation from a biomedical research setting to a clinical laboratory. In this review, we describe why and how glycomics is expected to find its role in clinical tests and the status of current mass spectrometry-based methods for clinical glycomics.

## 1 Introduction

There is a need for new biomarkers that target currently unmet clinical needs in patient diagnosis, stratification and monitoring. The proteome has long been recognized as a promising source of diagnostic markers and the growing understanding that a protein is actually a family of structurally variant molecules has impacted how we utilize this resource [1]. A vast diversity of proteoforms is caused by genetic and transcriptomic variation as well as post-translational modifications (PTMs) [2], [3]. One of the most abundant and complex PTMs is protein glycosylation, which involves the enzymatically regulated attachment of carbohydrate structures to proteins. This modification has been found to have a large disease biomarker potential [4]. Many proteins currently measured in the clinical laboratory are glycoproteins [5], [6], but there are only a few examples of glycosylation itself being used as a marker in clinical practice [7], [8], [9]. Many reviews on protein glycosylation have been published, often with a focus on disease processes [10], [11], [12], [13], [14]. Likewise, methods for protein glycosylation analysis have been covered [15], [16], [17], [18], [19]. In this review we aim to outline the path from the detection of glycan-based biomarker candidates to the development and implementation of medical tests. We will evaluate currently available analytical methods and approaches for protein glycosylation analysis, focusing on clinical applications, and discuss challenges that need to be addressed to promote the introduction of protein glycosylation tests in the medical laboratory.

### 1.1 Protein glycosylation

**Fig. 1:**
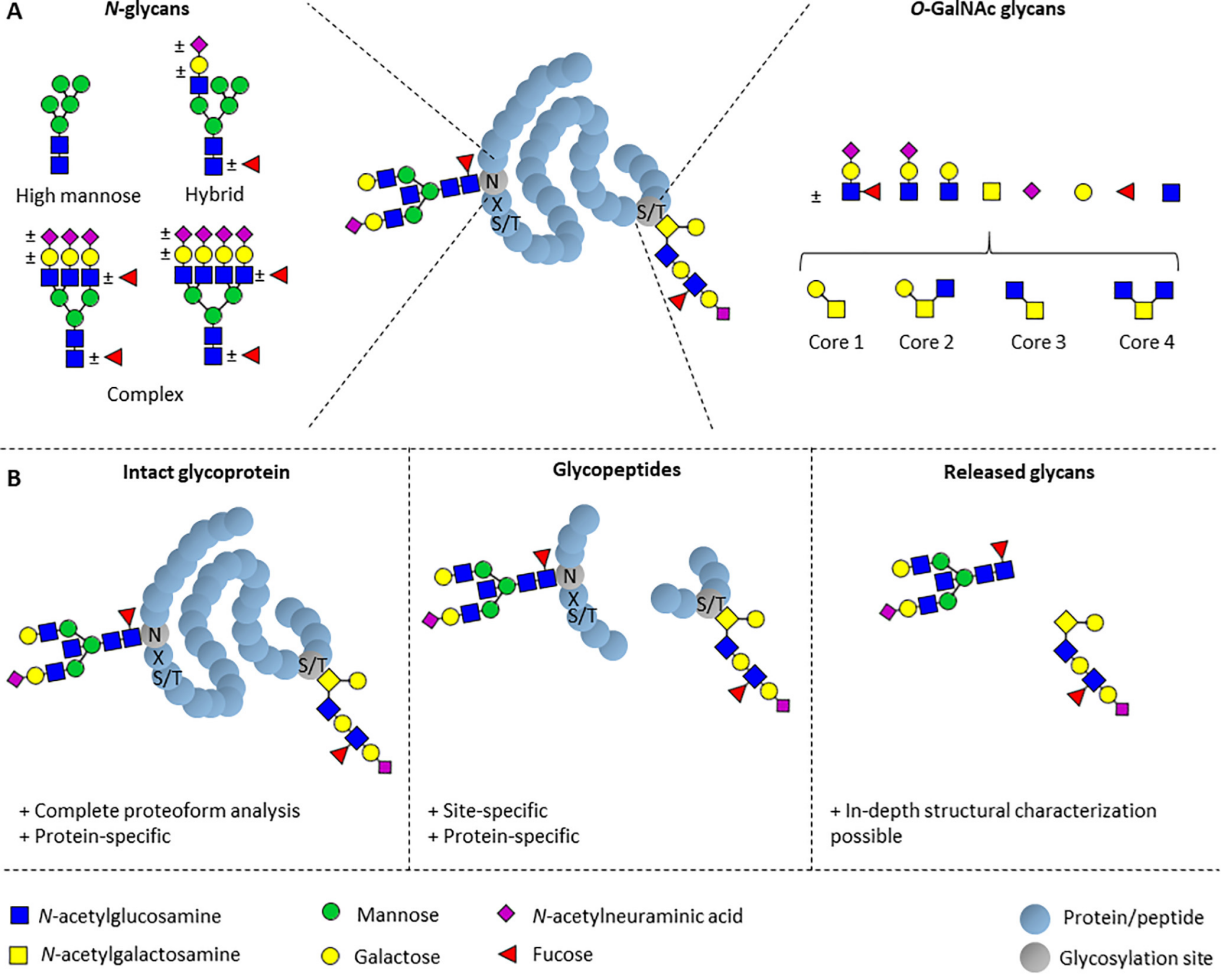
Glycosylation types most commonly found on human proteins and approaches for their analysis. (A) A selection of *N*- and *O*-GalNAc-glycans found on human proteins on their respective attachment sites [23]. (B) Analytical approaches to study protein glycosylation at the level of the intact glycoproteins, glycopeptides or released glycans and their specific advantages [17], [24]. Monosaccharides are depicted by symbols according to the Symbol Nomenclature for Glycans (SNFG) [25].

Over half of all human proteins are modified with one or more glycans [20]. These glycans can affect protein folding, stability, half-life, targeting, as well as receptor interaction [21]. Protein glycosylation is vastly heterogeneous and is accomplished by a range of different biosynthetic pathways. Two of the most abundant types of protein glycosylation are *N*-glycosylation and *O*-GalNAc glycosylation (*O*-glycosylation; Fig. 1A). *N*-glycosylation is initiated by the co- or post-translational transfer of a 14-monosaccharide precursor to the Asn in an Asn-Xxx-Ser/Thr (Xxx ≠ Pro) motif in the endoplasmic reticulum (ER) and subsequently subjected to glycosidase and glycosyltransferase treatment to reach a mature structure. *O*-glycosylation is initiated by the Golgi transfer of one GalNAc to a Ser or Thr residue after which enzymatic elongation and diversification occurs. While glycans are often attached to proteins, other glycoconjugates such as glycosphingolipids and glycosaminoglycans exist, and the overall set of glycan determinants covering the human glycome has been estimated to be many thousands [22]. The biosynthesis of protein and lipid glycosylation is a non-template-driven process and occurs in the ER and the Golgi apparatus by the interplay between glycan-modifying enzymes, sugar nucleotide transporters and monosaccharide donor availability. These processes are largely controlled locally and are, therefore, tissue- and cell type-specific. The six most prevalent monosaccharides in human protein glycosylation, as well as some examples of their involvement in the structures of mature glycans, are depicted in Fig. 1A. Many different glycan structures may occupy one protein glycosylation site causing diversity between protein copies (microheterogeneity). In addition, protein copies may carry multiple glycosylation sites with differences in site occupancy (macroheterogeneity). Combined, micro- and microheterogeneity lead to the presence of a large number of glycoforms of a protein. To reflect this heterogeneity, the analysis of glycoproteins usually does not target only one analyte, but rather a repertoire of glycoforms that together form the glycosylation profile of a protein, tissue or cell type. The structural variability as well as the potential engagement of glycoconjugates in glycan-protein interactions are, despite their importance, for the most part, still poorly explored [22].

### 1.2 Glycosylation changes in disease

Glycoconjugates play important roles in many physiological and pathophysiological processes [21]. Striking examples are a multitude of monogenic defects in the synthesis of glycoconjugates that can lead to very severe, rather rare disorders, the so-called congenital disorders of glycosylation (CDGs) [26], [27], [28]. CDGs are usually multi-organ pathologies and many variants result in prenatal death or severe cognitive impairment of the patient [26]. A genetic defect in one step in the glycan biosynthetic pathway often results in defect-specific glycosylation phenotypes that can be found throughout the glycoproteome of a CDG patient [29]. Due to these systemic phenotypes, a CDG often affects the integrity and function of multiple tissues. The versatility of CDG phenotypes illustrates the importance of a correct glycosylation in human development and homeostasis.

Next to CDGs, glycans are implicated in many prevalent human diseases [30]. This is reflected in up- or downregulation of certain glycoforms on specific proteins, resulting in altered glycosylation profiles. For various types of cancer, cell surface glycosylation is involved in disease processes, such as cell proliferation, metastasis and immune modulation [31]. Furthermore, the differential expression of glycoforms has been reported at both the cancer tissue level and the systemic level [31]. For example, serum immunoglobulins feature a reduced galactosylation in many cancer variants [32], [33], [34]. Also, in immune diseases and metabolic disorders, protein glycosylation is often affected [14], [33], [35], [36]. Of note, plasma protein glycosylation changes found in diabetes have recently received considerable attention as a potential source for novel biomarker and drug targets [35]. HNF1A-MODY-type diabetes is directly linked to the decrease of antennary fucosylation of plasma proteins, which has high potential to serve as a target in the diagnosis of this rare subtype of the disease [37]. While certain pathological conditions may result in vastly skewed protein glycosylation patterns, other factors such as genetic variation, sex, age, body mass index and smoking have also been described to induce considerable inter-person variability in the healthy population [14], [38]. A prominent example is the level of IgG galactosylation that correlates with sex and age, as well as overall health status [33]. This highlights the importance of taking these factors into account as potential confounding factors when studying glycans as disease markers.

## 2 Current status of clinical glycomics

Numerous clinical chemistry tests for protein levels used in patient diagnosis, stratification and monitoring target glycoproteins [6]. However, none of these assess glycoprotein-specific glycan features, but rather the overall glycoprotein concentration. Examples are prostate specific antigen (PSA), α-fetoprotein (AFP), carcinoembryonic antigen (CEA), mucin 1 (MUC1), MUC16, human epididymis protein 4 (HE4), human epidermal growth factor receptor 2 (HER2), thyroglobulin (Tg), many of the coagulation enzymes, and all immunoglobulin (Ig) isotypes [5], [6]. While most of these proteins have proven their applicability in diagnosing certain pathologies, e.g. the early detection of prostate cancer (PSA) or the monitoring of colorectal cancer treatment (CEA), they often lack clinical specificity [39], [40], [41]. This is partly caused by substantial inter-individual variation in protein levels in combination with an only moderate change in protein concentrations during the early stages of disease. Protein glycosylation, as described above, is a modification whose structure is highly dependent on the tissue and microenvironment where the glycoprotein is produced. Hence, it is thought that subpopulations of serum proteins, in the form of tumor tissue-specific glycoforms, may offer a higher specificity in disease diagnosis than the concentration of a protein alone [6]. Indications for improved clinical specificity through the quantitation of glycoforms have indeed been reported for, amongst others, the proteins PSA, AFP and IgG [33], [38], [42], [43]. For PSA glycans, higher levels of α2,3-linked sialylation are found in patients with high-risk prostate cancer [42], while for AFP glycosylation, the level of core fucosylation is positively associated with the occurrence of hepatocellular carcinoma and its progression [43]. Furthermore, IgG galactosylation levels, more than absolute IgG plasma levels, are found to be a marker for systemic inflammation [33], [38]. It must be noted that for most human proteins the glycosylation, as well as the clinical relevance thereof, has hitherto not been studied.

### 2.1 Glycomic markers in the clinic

While there are multiple reports of glycomic changes with disease, only a handful of glycomic markers are routinely used in the clinic [44]. For example, cancer antigen 19-9 (CA19-9) is a serum marker for monitoring response to therapy in patients with pancreatic adenocarcinoma [8]. This antigen is a tetrasaccharide (sialyl Lewis a), usually carried by glycolipids or mucins present in minor amounts in the circulation of healthy individuals. The increase of this antigen in the circulation is assessed in the clinical laboratory based on monoclonal antibody binding. Despite limitations, such as false negative test results in sialyl Lewis-negative individuals, CA19-9 remains an important glycomics marker for monitoring pancreatic cancer, as well as in the diagnosis of symptomatic patients [8].

In the case of hepatocellular carcinoma (HCC) and other liver conditions, clinical translation of glycomics biomarkers is already well-advanced [45], [46], [47]. Helena Biosciences has recently launched a glycomics-based blood test on a certified capillary electrophoresis (CE) platform for the diagnosis and prognosis of various liver diseases, including HCC [7]. The tests are based on total serum *N*-glycome profiles obtained by enzymatic release of glycans from the serum proteins. While the *N*-glycan release of total serum proteins does result in rather complex mixtures of hundreds of different structures [48], the clinically used method simplifies the challenge through enzymatic desialylation, followed by targeted quantification of just four glycoforms of interest [7], [45].

Mass spectrometry (MS) is an emerging technology in clinical glycomics. The most advanced example of clinical glycomics by MS is the analysis of transferrin glycosylation in the diagnosis of alcohol abuse and CDGs [26]. Carbohydrate deficient transferrin is routinely measured for alcohol abuse using immunonephelometric or immunoturbidimetric techniques [49], [50], but MS-based methods have been reported to characterize reference materials and calibrators [51]. Genetic analyses often play a key role in the diagnosis of CDGs, complemented by glycomics assays that include the intact mass analysis of human serum transferrin with its *N*-glycoforms [28]. In addition to its diagnostic power, MS analysis of transferrin glycoforms is instrumental in treatment monitoring [28]. A recent publication also attempted to define the type of CDG (i.e., identifying the enzyme affected) by quantifying specific glycans using multiple reaction monitoring (MRM)-MS [52]. Similar to CDGs, the degradation of glycoconjugates may be disturbed in monogenic diseases leading to lysosomal storage disorders [53]. These disorders are detected using an array of techniques, including genetic tests, enzyme assays and MS analysis of glycoconjugates from dried blood spots [54].

### 2.2 Clinical glycomics technology

In the past decade, MS has been recognized as a valuable technique for the quantification of proteins in the clinical chemistry laboratory [55]. Specifically, the use of MRM for targeted quantification of peptides representative of a protein for deducing protein concentrations was selected ‘Method of the Year’ by Nature Methods in 2012 [56]. The advantages of MS-based quantification of proteins over traditional immunoassays have been outlined clearly [57]. Immunoassays are fraught with a lack of concordance among immunoassay platforms, interference due to autoantibodies or anti-reagent antibodies and the high-dose hook effect. The direct quantification of proteins by MS, independent of antibodies, overcomes these disadvantages.

A second advantage of MS is its increased analytical specificity over conventional detection methods. MS-based methods allow for the unequivocal identification of glycans, peptides and glycopeptides through the unique combination of precursor mass and fragmentation patterns [58]. As such, the technique allows for the molecular characterization of proteins (the proteoforms), including the presence of specific glycoforms [17], [59]. The measurand of a test can, thus, be defined much more accurately using MS, as compared to using activity- or immunoassays, that are typically ‘blind’ to the individual proteins [3], [60].

Despite these advantages, current methods for glycomic analysis that have a high enough level of accuracy and precision to be used for clinical chemistry purposes rely predominantly on non-MS-based approaches. Important examples are antibody binding assays for the detection of specific glycan epitopes on intact glycoconjugates [8], [61] and CE with fluorescence detection (FLD) for the analysis of released and labeled *N*-glycans from plasma or serum [7], [45]. HILIC-FLD, likewise, has potential for highly robust analysis of released glycans in the clinic [62], but until now has not been used in this setting.

In contrast, MS is extensively used in glycomics biomarker discovery [17], particularly since the throughput and level of robustness that allows the glycosylation analysis of large numbers of clinical samples has recently been reached [63], [64]. This is important, because there is often not a single protein glycoform that shows biomarker potential for a certain condition, but rather a panel of structures that are, ideally, analyzed simultaneously [45]. It is anticipated that glycan-based markers that are discovered by MS methods could be validated and translated more easily if MS-based quantitative glycomics could be applied in clinical chemistry laboratories.

## 3 Mass spectrometric approaches for clinical glycomics

The translation of glycomic markers to the clinic, and the adaptation of MS for this purpose, is still in its infancy. Importantly, the requirements of methods for biomarker development, validation and implementation differ, as each of these phases requires the analysis of different numbers of samples, and a different level of accuracy and precision. For proteomics, this has resulted in the definition of 3 levels of methods [65]. Tier 3 methods may be used for biomarker discovery, with very limited requirements in terms of precision and accuracy. Tier 2 assays with more considerations for accuracy and precision can be used for biomarker validation. Tier 1 tests, that should fulfill stringent quality requirements, are needed for clinical chemistry purposes. Given the extreme complexity of protein glycosylation, most MS-based glycomics approaches are suitable for biomarker discovery studies, but their translation to widely used MS-based clinical chemistry tests requires further advancements.

All aspects of a test - the pre-analytical, analytical and post-analytical phases - should be well-designed during test development [66], and should already be considered in the early stages of biomarker discovery to avoid false positive results and research waste. The pre-analytical phase comprises sample collection, transport and storage. Glycomics studies have often been performed using serum or citrated plasma, yet it is unclear as to what extent glycomic signatures might change with storage conditions, and this should be evaluated in future studies. Recently, the use of dried blood spots as a stable matrix for the analysis of glycans has been reported [67], [68]. A number of critical elements can be identified that determine the quality of MS-based quantitative tests [66]. These include the definition of the measurand, the selection of the calibration strategy, the enzymatic digestion, as well as the selection of LC stationary phase, and MS detection mode. The Clinical Laboratory and Standards Institute (CLSI) has recently developed guidelines for the development of LC-MS-based quantitative tests [69], [70]. Once both the pre-analytical and analytical phases are in place, the definition of reference intervals or decision limits is important to allow accurate decision making and usability by clinicians. Guidance for the determination of these post-analytical parameters may be found in the CLSI document C28-A3c [71]. All the aspects mentioned above are important to ensure proper test performance once implemented into clinical care. To ensure that methods that are reported in literature can be reproduced and have been evaluated to a level that is accepted by the field, guidelines are often implemented for publication standards. Indeed, such guidelines have been described and should be adhered to for reporting of glycomic identifications [72], as well as clinical bottom-up proteomic methods [73].

Mass spectrometric protein glycosylation analysis may be performed at different levels, including the analysis of released glycans, glycopeptides and intact glycoproteins. Each of these approaches has their own strengths and weaknesses, resulting in their respective preferred applications (Fig. 1B) [17]. Of note, the protein- and site-specificity of glycosylation analysis achieved by analyzing intact glycoproteins or glycopeptides, often provides information that is functionally meaningful. For example, in the case of human IgG1, glycopeptide analysis allows specific assessment of the glycosylation of the IgG1 Fc portion. The results can be interpreted in light of the known influence of IgG1 Fc glycosylation on FcγIII-receptor interaction and resulting effector functions such as antibody-dependent cellular cytotoxicity [74].

**Fig. 2:**
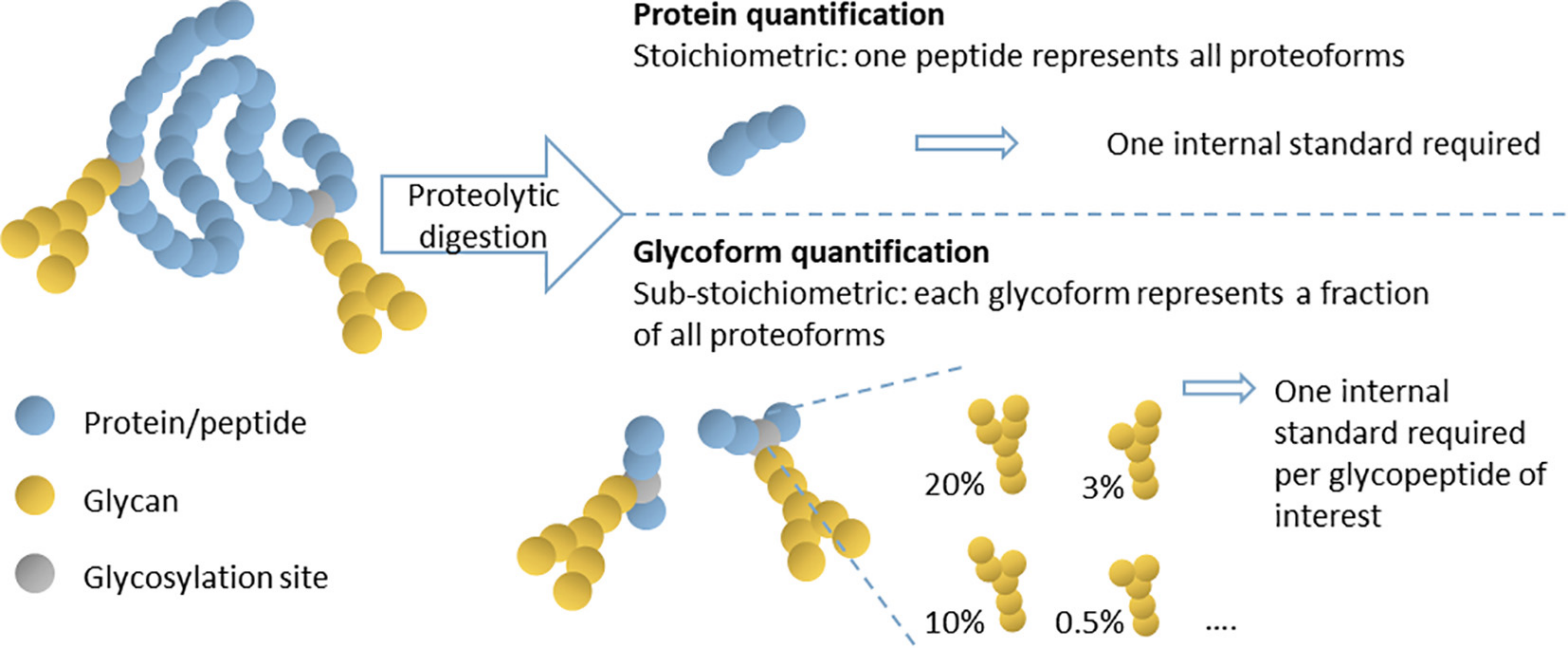
Sub-stoichiometric nature of glycosylation requires methods with high analytical sensitivity. While the MS-based quantification of proteins can rely on the detection of one peptide representative for all proteoforms, glycopeptide-based glycoform quantification requires the evaluation of one glycopeptide per glycoform of interest. Blue, yellow and grey balls give a schematic representation of a protein/peptide, glycan and glycosylation site, respectively. (For interpretation of the references to colour in this figure legend, the reader is referred to the web version of this article.)

One implication of the diverse and heterogeneous nature of glycosylation is that high analytical sensitivity is required. Where the assessment of protein concentrations by MS can rely on the analysis of one or two peptide(s) – representative of most, if not all, proteoforms – the quantification of a specific glycoform has to account for the sub-stoichiometric nature of glycosylation. It is not unusual that one protein glycoform accounts for less than 1% of the total abundance of a specific protein (Fig. 2).

### 3.1 Separation and ionization

In addition to their complexity and low abundance, glycans are relatively hydrophilic and do not carry a readily protonatable group, complicating their ionization in positive mode and desorption/desolvation, which is needed for MS analysis. This results in a reduced ionization efficiency for glycoconjugates, as compared to other analytes and matrix components, especially when released glycans or glycopeptides are assessed.

One approach to overcome the ion suppression of glycans or glycopeptides by their matrix components is to deplete the interferences in the samples [17]. This may be done via off-line solid-phase extraction (SPE) methods. Immunoaffinity or lectin enrichment are used when one is interested in specific glycoproteins or glycoforms, respectively. Hydrophilic interaction liquid chromatography (HILIC)-SPE is a tool for the untargeted enrichment of glycans and glycopeptides and is a common step in glycomics sample preparation [17]. However, for the introduction of glycomics in the clinical laboratory, enrichment steps are preferably omitted to reduce sample-handling and the introduction of biases.

Fortunately, glycosylation analysis by MS via electrospray ionization (ESI) has recently seen several developments that allow it to partially overcome the low ionization efficiency of glycans and glycopeptides via improved ionization regimes. A prominent example is the implementation of dopant enriched nitrogen-gas (DEN-gas) at the interface between a liquid-phase separation module (e.g., liquid chromatography (LC) or CE) and the MS [75], [76]. Using a DEN-gas setup, a vapor-enriched gas flows coaxially around the ESI emitter and enhances droplet desolvation [77]. This, in combination with the occurrence of higher charge states, has a positive effect on the detection of glycoconjugates [48], [75], [76]. Additionally, low-flow ESI conditions improve the analytical sensitivity of glycoconjugate analysis [78], [79]. Nano-LC and -CE setups with flow rates on the order of tens of nL/min have shown less discriminative ionization behaviors and, thus, higher sensitivities for glycoconjugates [79]. Yet, the nano-flow setups come with additional instrumental and robustness challenges, which may complicate their application in a clinical laboratory. The infrastructure required for the routine clinical assessment of protein glycosylation in the form of reversed-phase (RP)-LC-ESI-MS setups is appearing more commonly in clinical laboratories, some typical applications being newborn screening, hormone and drug analysis, as well as protein quantification [80]. RP-LC-MS is highly applicable for the analysis of intact glycoproteins and glycopeptides, as well as labeled glycans [17], [18], [81]. For the latter, RP-LC is even able to give more structural insights by resolving glycan isomers [18]. Alternative chromatographic and electrophoretic separation approaches, such as HILIC, porous graphitized carbon (PGC) and CE, have all shown their particular advantages in glycomic biomarker discovery, but are, as of yet, not commonly used in clinical practice. In this regard, we recently reported the development of a HILIC-MRM-MS based method for the separation of glycopeptides from PSA that would likely be suitable for clinical practice [82].

An attractive alternative to ESI for clinical glycomics by MS is provided by matrix-assisted laser desorption/ionization (MALDI), especially for the analysis of released glycans and glycopeptides [83]. MALDI-time-of-flight (TOF)-MS instruments are already established in clinical microbiology, where they are used for microbe identification via whole cell analysis [84]. MALDI-TOF-MS instrumentation is characterized by its ease of operation. Furthermore, while techniques relying on LC or CE separation are limited in their throughput, the omission of a separation module makes MALDI a high throughput alternative [17], [64], [85]. This technology has the potential to be of widespread use in laboratory medicine, outside of its well-established impact in medical microbiology. Though, as compared to ESI approaches coupled to LC or CE, MALDI-MS provides less information on glycosylation features, and the lability of sialylated glycoforms during ionization may limit its application. The latter issue can be resolved by sialic acid derivatization, although this results in more cumbersome sample preparation workflows [86]. Alternatively, in specific situations where sialylation is not of interest, glycoconjugates may be treated with neuraminidases to remove the sialic acids before subjecting them to MALDI, allowing straightforward glycan-feature detection. A similar approach was recently shown to be effective for the noninvasive diagnosis of liver cirrhosis and HCC [7], [45].

### 3.2 Mass spectrometric detection

**Fig. 3:**
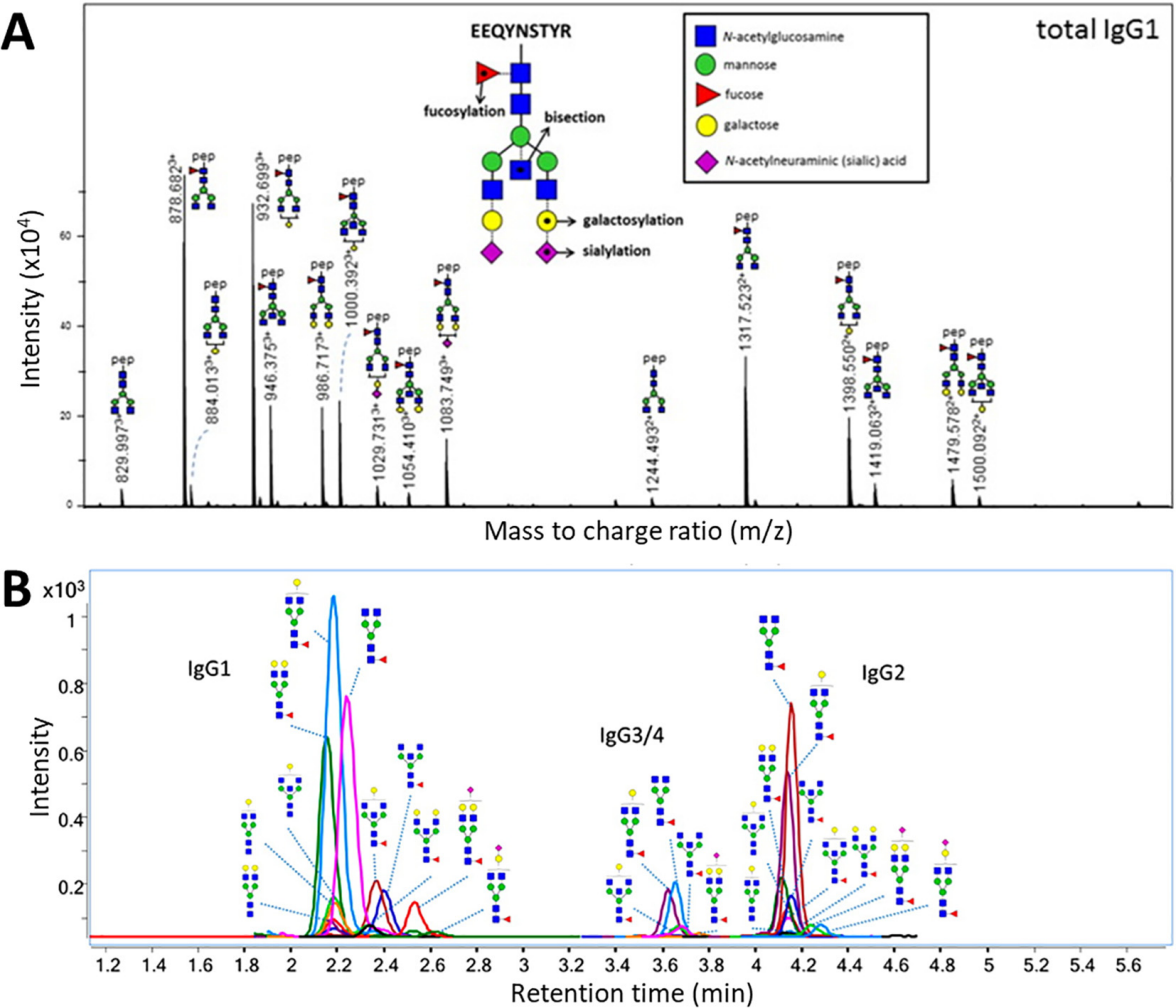
Characterization of immunoglobulin glycosylation. Immunoglobulin G (IgG) Fc glycopeptide analysis was performed in a discovery setting (A) using RP-LC-(q)TOF-MS, in which high resolution mass accuracy was achieved, and in a validation setting (B) using RP-LC-MRM-MS, which is better suitable for quantification in a clinical setting. Figures were taken from ([92], A) and ([93], B), with minor modifications.

Currently, most MS-based glycomics methods are used for (biomedical) research, for which other characteristics are required than for applications in medical laboratories. For research purposes, untargeted TOF- or iontrap-based analyses (using either ESI or MALDI) are often used, which allow for the identification of the complete repertoire of glycans or glycopeptides. An example of the LC-(q)TOF analysis of IgG glycopeptides is shown in Fig. 3A. Notably, while the collision induced dissociation (CID) fragmentation pattern of peptides is sequence-dependent, glycopeptides fragment primarily into mono-, di- and trisaccharide oxonium ions [87]. As a consequence, CID provides limited analytical specificity for glycopeptide identification, which hampers the identification of glycopeptides from complex mixtures through shotgun proteomics. While alternative fragmentation techniques, such as electron-transfer/higher-energy collision dissociation (EThcD) and ultraviolet photodissociation (UVPD) have been introduced on Orbitrap instruments [88], [89], [90], [91], these high-end mass spectrometers are not always readily available. Consequently, these MS/MS approaches are less commonly used, and it will require clear clinical applications of these techniques before their adoption in the medical laboratory may be considered.

In biomedical research, relative quantification of individual glycan structures or glycopeptides is typically performed by comparing signal intensity to the total glycan or glycopeptide signal intensity [94]. To address specific glycan features, glycan traits may be derived by calculating the ratios of groups of glycans with these traits, such as fucosylation, galactosylation or sialylation [86]. Both approaches are feasible in a research and biomarker discovery setting, but they require the quantification of all glycans in a sample or on a specific glycoprotein or glycopeptide. This is non-ideal for application in medical tests, where higher quality standards are required that should then hold up for all measured glycosylation features. For medical tests, the absolute quantification of individual glycans or glycoforms, relative to their labelled analogue would be a suitable alternative [95].

Traditionally, quantitative bottom-up proteomics strategies comprise the use of multiple reaction monitoring (MRM) on triple quadrupole mass spectrometers (QQQ-MS) as a targeted MS technique. Using this strategy clinical chemistry tests were already developed for several proteins [96], [97], [98] and, although substantial precautions need to be in place [99], robust performance of these tests could be shown [100]. In the larger and specialized laboratories, such instruments are, therefore, already available. Interestingly, both glycans [93], [101], [102] and glycopeptides [60], [93], [103] may also be quantified using MRM strategies [104], as illustrated in Fig. 3B. QQQ-MS instruments make use of CID fragmentation, and the oxonium ions are typically the glycan and glycopeptide fragments with highest intensity. However, these fragments are rather small and provide only limited analytical specificity, as almost all glycopeptides (except for high-mannose-type glycans) provide the same fragments in similar relative abundances. For glycopeptides, Y1 ions (consisting of the intact peptide and the innermost GlcNAc) may be monitored as an alternative, but, except for high-mannose type glycans, this results in loss of the (already poorer) analytical sensitivity of the quantification. While successful applications have been reported using MRM of glycopeptides by QQQ-MS [60], [82], [93], [105], alternative strategies with increased analytical specificity would be beneficial.

While the QQQ-MS is a low resolution instrument, improved analytical specificity for glycans and glycopeptides in targeted assays may be achieved through the use of higher resolution instrumentation such as TOF, Orbitrap or Fourier transform ion cyclotron resonance (FTICR)-MS [106]. Indeed, the use of targeted quantification of proteins using high-resolution, accurate-mass MS is termed parallel reaction monitoring (PRM) [107], [108]. The use of PRM substantially increases the resolution of the transitions monitored, and could improve the analytical specificity of targeted glycopeptide quantification [109]. However, the linear dynamic range of the Orbitrap-MS remains to be compared to QQQ-MS for quantitative purposes, and it should be noted that Orbitraps are high-end mass spectrometers that are only available in specialized laboratories.

**Fig. 4:**
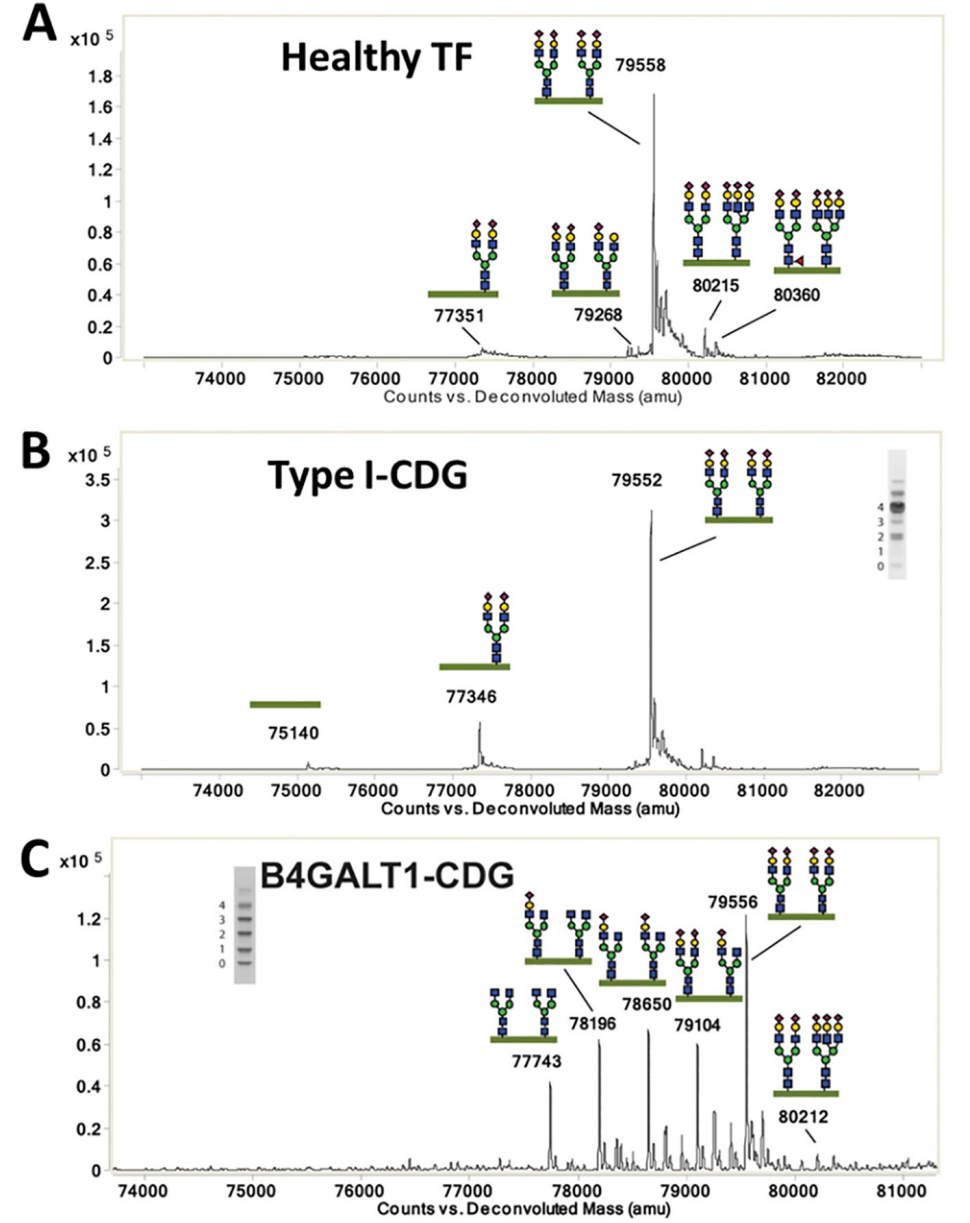
Intact glycoprotein analysis by nRP-LC-TOF-MS reveals differential transferrin glycosylation for patients with congenital disorders of glycosylation (CDGs). Deconvoluted intact protein mass spectra of transferrin from plasma from (A) a healthy volunteer, revealed the presence of two glycans, mostly diantennary and fully sialylated each. (B) A mild type I CDG revealed the lack of a full glycan, while (C) a type II CDG showed much higher glycoform variability. Figures were taken from [9], with modifications.

Besides the analysis of glycans and glycopeptides, protein glycosylation may also be assessed using intact glycoprotein analysis. A clear advantage of this method is that mass information on the full protein is obtained, including all sites of glycosylation (Fig. 1). Different than for glycopeptides, the glycan fraction of a glycoprotein is relatively small, which reduces the ionization bias when analyzing intact glycoproteins. Another advantage of an intact glycoprotein analysis in a clinical laboratory is the limited sample preparation required prior to MS detection. Usually, a simple affinity- or immune- purification of the protein of interest is sufficient for its glycomic characterization. Limiting factors in the implementation of intact glycoprotein analysis could include advanced technical requirements regarding analytical sensitivity and resolution, and the complexity of the data analysis. Despite these challenges, the intact analysis of the glycoprotein transferrin by C8-RP-HPLC coupled to high resolution (HR)-TOF-MS has emerged as an invaluable tool in the detection and differentiation of CDGs (Fig. 4) [9]. Provided that high-resolution mass spectrometers become more widely available, intact glycoprotein analysis has the potential to become feasible for clinical applications.

### 3.3 Internal standardization and calibration

As indicated previously, the requirements of tests suitable for clinical chemistry implementation are more stringent than those used for biomedical research and biomarker discovery [65]. Internal standardization and calibration may not yet be necessary for biomarker discovery studies using Tier 3 methods [103]. However, internal standardization should be used during biomarker validation studies to achieve better analytical specificity and quantitative results in Tier 2 assays. Recently, a non-MS based method for translation of glycomics markers was reported [110]. It is imperative for successful clinical chemistry test development that each individual test result is accurate and reproducible and, thus, a Tier 1 test, including internal standardization and accurate calibration is required.

The development of effective calibration methodology is vital for successful establishment of reproducible glyco-diagnostics in a clinical setting. Because MS is inherently not a quantitative technique and signal intensity is influenced by matrix-dependent ion suppression [111], absolute quantification relies on the availability of stable isotope labeled internal standards [112]. While the synthesis of peptides, including incorporation of ^13^C,^15^N-stable isotope labeled amino acids, is now routinely performed, the synthesis of glycoconjugates is much more difficult. Particularly, the synthesis of specific glycan linkage isomers was achieved only recently [113], [114]. However, pure ^13^C labeled free glycans are now available from a number of commercial sources, and have been shown to improve quantification of individual glycoforms in released glycan approaches [115]. Because glycans are often chemically derivatized prior to their analysis [116], isotope labels can also easily be incorporated during derivatization [117], [118]. A more dire situation exists at the level of glycopeptides and intact glycoproteins. The synthesis of pure glycopeptides or glycoproteins with full length labeled glycans is not (yet) possible. The synthetic coupling of a glycan to an asparagine residue, either as a single amino acid or incorporated into a peptide, remains a challenging task. However, synthetic peptides carrying a single GlcNAc at the asparagine residue have been reported [119]. Similarly, a mouse monoclonal antibody has been produced recombinantly, which carries full-length ^15^N labeled glycans [120] as well as glycosylated human monoclonal antibodies with heavy labeled lysines and arginines incorporated in the protein backbone [121]. While these initial reports are very promising, highly pure glycopeptides and glycoproteins carrying a single glycan structure would be desirable.

Besides internal standards, a suitable calibration strategy should also comprise well characterized and accurately quantified external calibrators. Ideally, these calibrators should be traceable to SI to enable standardization and worldwide comparability of results. However, while this is already a tour-de-force in the field of protein quantification [122], [123], [124], the added complexity of glycosylation makes this a daunting task. Indeed, in a study by NIST on the glycomic characterization of an antibody using different techniques in 76 laboratories (all characterized to a level for biomedical research and not for clinical chemistry), variable results were obtained [125]. This was partly due to the variability in analytical sensitivity and resolution between methods and different ionization efficiencies in the MS-based technologies [126]. Despite the differences, a consensus relative abundance could be calculated for 57 glycoforms, which may form the basis for the well-needed harmonization of glycosylation analysis techniques. A working group from the international federation of clinical chemistry (IFCC) was dedicated to the standardization of the measurement of carbohydrate deficient transferrin (CDT). Within this working group, a reference material based on human serum was established in which the percentage disialotransferrin to total transferrin fraction was determined within a reference laboratory network [127], [128]. The cited examples demonstrate that it is possible to produce calibration markers of relative glycan abundances, but calibrators of intact protein glycoforms or glycopeptides with demonstrable SI units are yet to be developed. In this light, a recent attempt to purify and quantify a specific glycopeptide as a calibrator for absolute quantification of glycopeptides, should be an encouragement [129]. However, value assignment of this glycopeptide was not traceable, the production was costly, material is not widely available and purification would be necessary for each individual glycopeptide. The situation is different if analysis only at the glycan level is required, as techniques for the characterization and quantification of glycans are available. Similarly to peptides, the purity of glycans may be assessed using MS-based compositional [130], [131] and linkage analysis [132], [133], [134]. For quantification, glycans may be hydrolyzed to monosaccharides [135], or subjected to NMR spectroscopy [136].

## 4 Enzymatic digestion

In bottom-up proteomics, which is typically used for the quantification of proteins and glycoproteins by MS, the protein is enzymatically cleaved into the peptides and glycopeptides that are quantified. Because the actual measurand is changed from the intact glycoprotein into peptides and glycopeptides, it is important that the digestion kinetics, preferably, but even more importantly, the final (glyco)peptide yield are constant, independent of the matrix and glycan involved. Conditions that should be considered are the digestion buffer and denaturing agents, as well as the aid of protein-binding matrices such as S-trap and FASP [137], [138], [139]. For peptides, this has resulted in several in-depth studies towards optimal digestion conditions and the digestion kinetics in relation to peptide selection [140]. For glycopeptides, a complicating factor is that specific glycoforms may affect the digestion efficiency. Indeed, the presence of a large number of *O*-glycans on mucins has already been reported to hamper mucin digestion [141]. Similarly, in a recent study in which digestion conditions for IgG *N*-linked glycans were studied, a strong preferential digestion of high mannose, hybrid, alpha2-3-sialylated and bisected glycoforms was observed [142]. It was also reported that fucosylated glycans in close proximity to the proteolytic cleavage site may hamper the digestion [143]. Digestion biases could be partly resolved under denaturing conditions, but indicate that digestion conditions should be carefully selected and monitored to ensure robust glycopeptide quantification.

## 5 Other considerations for the development of high-quality clinical glycomics tests for the medical laboratory

Besides analytical challenges, the translation of glycomics research into actionable clinical parameters is also hampered by a lack of clear clinical evidence. While strong efforts are made to draft dedicated study designs, including hypotheses, cohort selection and method selection, it should be stressed that these should ideally be guided by well-specified unmet clinical needs [144], [145]. For the successful translation of fundamental glycomic research to the clinical laboratory, and the incorporation of the developed tests in clinical practice, an interaction triangle is warranted between biomarker developers, clinical chemists, and clinicians. These three parties should collaborate to define the unmet clinical need and direct the preclinical research towards actionable results that fulfill clinical performance criteria [146], [147]. An early stage partnership between the three parties prevents the accumulation of glycomic associations with diseases in the pre-clinical phase, without perspective of reaching daily clinical practice.

One reason for the limited translation of the now reported glycomics markers to the clinic is the lack of replication of the performance of these markers. This is a challenge encountered also in other fields, such as metabolomics and proteomics [148], [149], [150], and is partly caused by the shortage of large and well-defined prospective clinical cohorts (or their limited accessibility) and an insufficient statistical evaluation of the initial findings, resulting in poor validation of initial results. An example would be the reporting of 8 differentially expressed *N*-glycans in 82 breast cancer patients compared to 27 healthy controls in one study [151], while a different study reported 25 different *N*-glycans to be differentially expressed in 256 breast cancer patients compared to 311 healthy controls [152]. While the analytical methods used were different, it is highly likely that these incongruent results are confounded by general descriptors of the population, such as age, sex and BMI [14], [38], [153]. Additionally, even one glycoprotein can already occur in dozens or even hundreds of glycoforms, drastically increasing the number of variables in an exploratory study. To limit the number of false positive assignments, multiple testing correction is essential and has to be considered in the power assessments. Equally important are the inclusion and definition of different patient groups, for which differential diagnosis and treatment is relevant. Ultimately, exploratory glycomic research for a well-defined clinical question should result in a model including a subset of glycoforms that shows predictive value for a disease. Of note, development and registration of such a glycoform pattern or signature will often require advanced biostatistical and bioinformatics approaches. Replication in an independent sample set is essential to assure its translational potential. For successful clinical implementation the simplicity and robustness of such models and algorithms will be of utmost importance, and the outcome of the test has to be presented in a simple and comprehensible manner in order facilitate acceptance by clinicians.

To ensure that reasonable expectations are set for each of the stakeholders in the medical test development pipeline, it is imperative that everyone is aware of the possibilities and limitations of current glyco-analytical technologies. Glycobiology is an emerging and highly interdisciplinary field, currently not commonly included in (bio)medical university curricula [154]. It involves knowledge on the chemistry of carbohydrates, enzymology for glycan formation and processing, the role of glycans in systems biology and techniques to characterize and manipulate the glycosylation of a living system. To enhance communication and mutual understanding between the three parties, efforts should be taken by fundamental glyco-scientists to disseminate glycobiological and glyco-analytical knowledge to medical professionals, clinical chemists and biomedical researchers, in line with recently published recommendations [154].

## 6 Conclusions and future perspectives

Over the past decades, tremendous improvements have been made in analytical technology for the identification and quantification of protein glycosylation, which now make the translation of such technologies into clinical practice feasible. Specifically, methodologies are now in place to perform glycosylation analyses in a high-throughput and sufficiently robust [155] manner for biomarker discovery. To enable translation of biomarkers from discovery through validation into clinical tests, analytical methods that are fit-for-purpose, with increased levels of accuracy and precision are required [65]. To truly enable translation of glycomics-based tests, further investigations into the preanalytical requirements, as well as digestion conditions are needed. The development of stable internal standards and calibrators that reflect and represent the endogenous protein glycosylation is also needed for improved robustness, precision and accuracy. An interesting observation is the relative intra-individual stability of the human glycome in the absence of major physiological or pathological changes [156], [157]. Longitudinal monitoring of protein glycosylation could have the potential to reveal pathophysiological changes at an early stage if repeated sampling is employed. Such a strategy would allow for the use of reference change values [158], [159], compared to reference intervals or decision limits.

It is now more and more emphasized that the analytical rigor of methods and tests used not only for diagnostics, but already during biomarker development should be of high standard and well documented [160], [161]. Recently, standards were developed for reporting the use of clinical bottom-up proteomics methods in scientific literature [73]. While guidelines for reporting glycomic identifications have also been described [72], it should be emphasized that a guideline on the information needed for reporting clinical glycomics data for application in diagnostics should be developed.

Clinical chemistry tests should allow for absolute quantification of (glyco)proteins. Ideally, equivalence of test results in time and space is achieved through metrological traceability to SI units. Worldwide standardization efforts are in place to develop reference measurement systems to accomplish this at the protein level. However, the current end-user measuring systems (whether as a *lab-developed-test* or commercially available) often do not take protein glycosylation into account [3], [60], and are unaware of potential interference due to differential glycosylation. It is, thus, important to know the clinical implications of glycosylation that may be present on current diagnostic markers. A proper definition of the measurand, as is currently being attempted for antithrombin [104], is highly beneficial for standardization efforts.

As presented in this review, there is ample evidence that protein glycosylation plays pivotal roles in the onset and progression of diseases. Direct evidence for the utility of protein glycosylation to resolve unmet clinical needs remains sparse. However, the rapidly developing analytical technologies and large and well-designed glycomics studies specifically addressing such well-defined unmet clinical needs are likely to accelerate the role of glycomics in clinical test development in the near future.

## Funding

This work was partially funded by an EU H2020 MSCA individual fellowship #843615 to LRR.

## Declaration of Competing Interest

The authors declare that they have no known competing financial interests or personal relationships that could have appeared to influence the work reported in this paper.

## References

[1] Geyer P.E. (2017). Revisiting biomarker discovery by plasma proteomics. Mol. Syst. Biol..

[2] L.M. Smith, N.L. Kelleher, P. Consortium for Top Down, Proteoform: a single term describing protein complexity, Nat. Methods 10(3) (2013) 186–187. 10.1038/nmeth.2369 PMC4114032 23443629

[3] van der Burgt Y.E.M., Cobbaert C.M. (2018). Proteoform analysis to fulfill unmet clinical needs and reach global standardization of protein measurands in clinical chemistry proteomics. Clin. Lab. Med..

[4] N.H. Packer, et al., Frontiers in glycomics: bioinformatics and biomarkers in disease. An NIH white paper prepared from discussions by the focus groups at a workshop on the NIH campus, Bethesda MD (September 11–13, 2006). Proteomics 8(1) (2008) 8–20. 10.1002/pmic.200700917 18095367

[5] Kirwan A. (2015). Glycosylation-based serum biomarkers for cancer diagnostics and prognostics. Biomed. Res. Int..

[6] Schulz B.L., Laroy W., Callewaert N. (2007). Clinical laboratory testing in human medicine based on the detection of glycoconjugates. Curr. Mol. Med..

[7] H.B. Europe, Helena biosciences: Glyco Liver Profile. [cited 2019; Available from: http://www.helena-biosciences.com/en/clinical-electrophoresis/v8-nexus/tests/glyco-liver-profile/.

[8] Scara S., Bottoni P., Scatena R. (2015). CA 19–9: biochemical and clinical aspects. Adv. Exp. Med. Biol..

[9] van Scherpenzeel M. (2015). High-resolution mass spectrometry glycoprofiling of intact transferrin for diagnosis and subtype identification in the congenital disorders of glycosylation. Transl. Res..

[10] Clerc F. (2016). Human plasma protein N-glycosylation. Glycoconj. J..

[11] Munkley J., Elliott D.J. (2016). Hallmarks of glycosylation in cancer. Oncotarget.

[12] Stowell S.R., Ju T., Cummings R.D. (2015). Protein glycosylation in cancer. Annu. Rev. Pathol..

[13] Maverakis E. (2015). Glycans in the immune system and the altered glycan theory of autoimmunity: a critical review. J. Autoimmun..

[14] Dotz V., Wuhrer M. (2019). N-glycome signatures in human plasma: associations with physiology and major diseases. FEBS Lett..

[15] Xiao H. (2019). Global and site-specific analysis of protein glycosylation in complex biological systems with Mass Spectrometry. Mass Spectrom. Rev..

[16] Kailemia M.J. (2014). Oligosaccharide analysis by mass spectrometry: a review of recent developments. Anal. Chem..

[17] Ruhaak L.R. (2018). Mass spectrometry approaches to glycomic and glycoproteomic analyses. Chem. Rev..

[18] Vreeker G.C., Wuhrer M. (2017). Reversed-phase separation methods for glycan analysis. Anal. Bioanal. Chem..

[19] Stavenhagen K., Kolarich D., Wuhrer M. (2015). Clinical glycomics employing graphitized carbon liquid chromatography-mass spectrometry. Chromatographia.

[20] Khoury G.A., Baliban R.C., Floudas C.A. (2011). Proteome-wide post-translational modification statistics: frequency analysis and curation of the swiss-prot database. Sci. Rep..

[21] Varki A. (2017). Biological roles of glycans. Glycobiology.

[22] Cummings R.D. (2009). The repertoire of glycan determinants in the human glycome. Mol. BioSyst..

[23] A. Varki, S. Kornfeld, Historical background and overview, in: Essentials of Glycobiology, rd, et al., Editors, 2015, Cold Spring Harbor (NY), pp. 1–18.

[24] Marino K. (2010). A systematic approach to protein glycosylation analysis: a path through the maze. Nat. Chem. Biol..

[25] Neelamegham S. (2019). Updates to the symbol nomenclature for glycans guidelines. Glycobiology.

[26] Peanne R. (2018). Congenital disorders of glycosylation (CDG): Quo vadis?. Eur. J. Med. Genet..

[27] Saldova R. (2015). N-glycosylation of serum IgG and total glycoproteins in MAN1B1 deficiency. J. Proteome Res..

[28] Van Scherpenzeel M., Willems E., Lefeber D.J. (2016). Clinical diagnostics and therapy monitoring in the congenital disorders of glycosylation. Glycoconj. J..

[29] Abu Bakar N., Lefeber D.J., van Scherpenzeel M. (2018). Clinical glycomics for the diagnosis of congenital disorders of glycosylation. J. Inherit. Metab. Dis..

[30] N.R. Council, Transforming Glycoscience: A Roadmap for the Future, National Academies Press, 2012. 23270009

[31] Mereiter S. (2019). Glycosylation in the era of cancer-targeted therapy: where are we heading?. Cancer Cell.

[32] Adamczyk B., Tharmalingam T., Rudd P.M. (2012). Glycans as cancer biomarkers. BBA.

[33] Gudelj I., Lauc G., Pezer M. (2018). Immunoglobulin G glycosylation in aging and diseases. Cell. Immunol..

[34] Pinho S.S., Reis C.A. (2015). Glycosylation in cancer: mechanisms and clinical implications. Nat. Rev. Cancer.

[35] Rudman N., Gornik O., Lauc G. (2019). Altered N-glycosylation profiles as potential biomarkers and drug targets in diabetes. FEBS Lett..

[36] Lu L.L. (2016). A functional role for antibodies in tuberculosis. Cell.

[37] Juszczak A. (2019). Plasma fucosylated glycans and C-reactive protein as biomarkers of HNF1A-MODY in young adult-onset nonautoimmune diabetes. Diab. Care.

[38] Plomp R. (2017). Subclass-specific IgG glycosylation is associated with markers of inflammation and metabolic health. Sci. Rep..

[39] Nicholson B.D. (2015). Blood CEA levels for detecting recurrent colorectal cancer. Cochrane Database Syst. Rev..

[40] Thompson I.M. (2004). Prevalence of prostate cancer among men with a prostate-specific antigen level < or =4.0 ng per milliliter. N. Engl. J. Med..

[41] The PSA position (2011). Nature.

[42] Llop E. (2016). Improvement of prostate cancer diagnosis by detecting PSA glycosylation-specific changes. Theranostics.

[43] Tada T. (2005). Relationship between Lens culinaris agglutinin-reactive alpha-fetoprotein and pathologic features of hepatocellular carcinoma. Liver Int..

[44] Almeida A., Kolarich D. (2016). The promise of protein glycosylation for personalised medicine. BBA.

[45] Callewaert N. (2004). Noninvasive diagnosis of liver cirrhosis using DNA sequencer-based total serum protein glycomics. Nat. Med..

[46] de Oliveira R.M., Ornelas Ricart C.A., Araujo Martins A.M. (2017). Use of mass spectrometry to screen glycan early markers in hepatocellular carcinoma. Front. Oncol..

[47] Comunale M.A. (2013). Total serum glycan analysis is superior to lectin-FLISA for the early detection of hepatocellular carcinoma. Proteomics Clin. Appl..

[48] Lageveen-Kammeijer G.S.M. (2019). Highly sensitive CE-ESI-MS analysis of N-glycans from complex biological samples. Nat. Commun..

[49] Bortolotti F. (2018). Analytical and diagnostic aspects of carbohydrate deficient transferrin (CDT): a critical review over years 2007–2017. J. Pharm. Biomed. Anal..

[50] Nomura F. (2018). Determination of serum carbohydrate-deficient transferrin by a nephelometric immunoassay for differential diagnosis of alcoholic and non-alcoholic liver diseases. Clin. Chim. Acta.

[51] Oberrauch W., Bergman A.C., Helander A. (2008). HPLC and mass spectrometric characterization of a candidate reference material for the alcohol biomarker carbohydrate-deficient transferrin (CDT). Clin. Chim. Acta.

[52] He M. (2019). Increased clinical sensitivity and specificity of plasma protein N-glycan profiling for diagnosing congenital disorders of glycosylation by use of flow injection-electrospray ionization–quadrupole time-of-flight mass spectrometry. Clin. Chem..

[53] Parenti G., Andria G., Ballabio A. (2015). Lysosomal storage diseases: from pathophysiology to therapy. Annu. Rev. Med..

[54] Wang R.Y. (2011). Lysosomal storage diseases: diagnostic confirmation and management of presymptomatic individuals. Genet. Med..

[55] Lehmann S. (2013). Quantitative Clinical Chemistry Proteomics (qCCP) using mass spectrometry: general characteristics and application. Clin. Chem. Lab. Med..

[56] Method of the Year 2012, Nat. Methods 10(1) (2013) 1. 10.1038/nmeth.2329 23547284

[57] Hoofnagle A.N., Wener M.H. (2009). The fundamental flaws of immunoassays and potential solutions using tandem mass spectrometry. J. Immunol. Methods.

[58] Matthiesen R., Bunkenborg J. (2013). Introduction to mass spectrometry-based proteomics. Methods Mol. Biol..

[59] Schaffer L.V. (2019). Identification and quantification of proteoforms by mass spectrometry. Proteomics.

[60] Ruhaak L.R. (2018). Detecting molecular forms of antithrombin by LC-MRM-MS: defining the measurands. Clin. Chem. Lab. Med..

[61] Yoneyama T. (2014). Measurement of aberrant glycosylation of prostate specific antigen can improve specificity in early detection of prostate cancer. Biochem. Biophys. Res. Commun..

[62] Colhoun H.O. (2018). Validation of an automated ultraperformance liquid chromatography IgG N-glycan analytical method applicable to classical galactosaemia. Ann. Clin. Biochem..

[63] Bakovic M.P. (2013). High-throughput IgG Fc N-glycosylation profiling by mass spectrometry of glycopeptides. J. Proteome Res..

[64] Reiding K.R. (2019). High-throughput serum N-glycomics: method comparison and application to study rheumatoid arthritis and pregnancy-associated changes. Mol. Cell. Proteomics.

[65] Carr S.A. (2014). Targeted peptide measurements in biology and medicine: best practices for mass spectrometry- based assay development using a fit- for- purpose approach. Mol. Cell. Proteomics.

[66] Smit N. (2014). Quality requirements for quantitative clinical chemistry proteomics. Transl. Proteomics.

[67] Ruhaak L.R. (2012). N-Glycan profiling of dried blood spots. Anal. Chem..

[68] Vreeker G.C.M. (2019). Dried blood spot N-glycome analysis by MALDI mass spectrometry. Talanta.

[69] Lynch K.L. (2016). CLSI C62-A: a new standard for clinical mass spectrometry. Clin. Chem..

[70] C.a.L.S. Institute, C62-A. Liquid Chromatography-Mass Spectrometry Methods; Approved Guideline, 2014.

[71] G.L. Horowitz, et al., Defining, Establishing, and Verifying Reference Intervals in the Clinical Laboratory: Approved Guideline, 3rd ed., C28-A3c, Vol. 28. Clinical & Laboratory Standards Institute, 2008, p. 61.

[72] York W.S. (2014). MIRAGE: the minimum information required for a glycomics experiment. Glycobiology.

[73] Vogeser M., Schuster C., Rockwood A.L. (2019). A proposal to standardize the description of LC–MS-based measurement methods in laboratory medicine. Clin. Mass Spectrometry.

[74] Dekkers G. (2017). Decoding the human immunoglobulin G-glycan repertoire reveals a spectrum of Fc-receptor- and complement-mediated-effector activities. Front. Immunol..

[75] Falck D. (2017). High-throughput analysis of IgG Fc glycopeptides by LC-MS. Methods Mol. Biol..

[76] Kammeijer G.S. (2016). Dopant enriched nitrogen gas combined with sheathless capillary electrophoresis-electrospray ionization-mass spectrometry for improved sensitivity and repeatability in glycopeptide analysis. Anal. Chem..

[77] Nguyen S., Fenn J.B. (2007). Gas-phase ions of solute species from charged droplets of solutions. Proc. Natl. Acad. Sci. USA.

[78] Busnel J.M. (2010). High capacity capillary electrophoresis-electrospray ionization mass spectrometry: coupling a porous sheathless interface with transient-isotachophoresis. Anal. Chem..

[79] Juraschek R., Dulcks T., Karas M. (1999). Nanoelectrospray–more than just a minimized-flow electrospray ionization source. J. Am. Soc. Mass Spectrom..

[80] Jannetto P.J., Fitzgerald R.L. (2016). Effective use of mass spectrometry in the clinical laboratory. Clin. Chem..

[81] Camperi J., Pichon V., Delaunay N. (2019). Separation methods hyphenated to mass spectrometry for the characterization of the protein glycosylation at the intact level. J. Pharm. Biomed. Anal..

[82] van der Burgt Y.E.M. (2020). HILIC-MRM-MS for linkage-specific separation of sialylated glycopeptides to quantify prostate-specific antigen proteoforms. J. Proteome Res..

[83] Harvey D.J. (2018). Analysis of carbohydrates and glycoconjugates by matrix-assisted laser desorption/ionization mass spectrometry: an update for 2013–2014. Mass Spectrom. Rev..

[84] Patel R. (2013). MALDI-TOF mass spectrometry: transformative proteomics for clinical microbiology. Clin. Chem..

[85] Reusch D. (2015). Comparison of methods for the analysis of therapeutic immunoglobulin G Fc-glycosylation profiles-Part 2: mass spectrometric methods. MAbs.

[86] Bladergroen M.R. (2015). Automation of high-throughput mass spectrometry-based plasma N-glycome analysis with linkage-specific sialic acid esterification. J. Proteome Res..

[87] An H.J., Lebrilla C.B. (2011). Structure elucidation of native N- and O-linked glycans by tandem mass spectrometry (tutorial). Mass Spectrom. Rev..

[88] Cotham V.C., Brodbelt J.S. (2016). Characterization of therapeutic monoclonal antibodies at the subunit-level using middle-down 193 nm ultraviolet photodissociation. Anal. Chem..

[89] Ko B.J., Brodbelt J.S. (2015). Comparison of glycopeptide fragmentation by collision induced dissociation and ultraviolet photodissociation. Int. J. Mass Spectrom..

[90] Halim M.A. (2018). Ultraviolet, infrared, and high-low energy photodissociation of post-translationally modified peptides. J. Am. Soc. Mass Spectrom..

[91] Reiding K.R. (2018). The benefits of hybrid fragmentation methods for glycoproteomics. TrAC, Trends Anal. Chem..

[92] Kemna M.J. (2017). Galactosylation and sialylation levels of IgG predict relapse in patients With PR3-ANCA associated Vasculitis. EBioMedicine.

[93] Hong Q. (2013). Absolute quantitation of immunoglobulin G and its glycoforms using multiple reaction monitoring. Anal. Chem..

[94] Benedetti E. (2019). Systematic evaluation of normalization methods for glycomics data based on performance of network inference. bioRxiv.

[95] Etxebarria J., Reichardt N.C. (2016). Methods for the absolute quantification of N-glycan biomarkers. BBA.

[96] van den Broek I. (2016). Automated multiplex LC-MS/MS assay for quantifying serum apolipoproteins A-I, B, C-I, C-II, C-III, and E with qualitative apolipoprotein E phenotyping. Clin. Chem..

[97] Kushnir M.M. (2013). Measurement of thyroglobulin by liquid chromatography-tandem mass spectrometry in serum and plasma in the presence of antithyroglobulin autoantibodies. Clin. Chem..

[98] Lehmann S. (2017). Clinical mass spectrometry proteomics (cMSP) for medical laboratory: What does the future hold?. Clin. Chim. Acta.

[99] M. Vogeser, Mass spectrometry in the clinical laboratory - challenges for quality assurance, in: Current Trends in Mass Spectrometry, 2015, pp. 14–19.

[100] Ruhaak L.R. (2018). Robust and accurate 2-year performance of a quantitative mass spectrometry-based apolipoprotein test in a clinical chemistry laboratory. Clin. Chem..

[101] Zhou S. (2015). Quantitation of permethylated N-glycans through multiple-reaction monitoring (MRM) LC-MS/MS. J. Am. Soc. Mass Spectrom..

[102] Wang J.-R. (2017). A method to identify trace sulfated IgG N-glycans as biomarkers for rheumatoid arthritis. Nat. Commun..

[103] Miyamoto S. (2018). Multiple reaction monitoring for the quantitation of serum protein glycosylation profiles: application to ovarian cancer. J. Proteome Res..

[104] Ruhaak L.R. (2017). The use of multiple reaction monitoring on QQQ-MS for the analysis of protein- and site-specific glycosylation patterns in serum. Methods Mol. Biol..

[105] Song E., Pyreddy S., Mechref Y. (2012). Quantification of glycopeptides by multiple reaction monitoring liquid chromatography/tandem mass spectrometry. Rapid Commun. Mass Spectrom..

[106] Ruhaak L.R., van der Burgt Y.E., Cobbaert C.M. (2016). Prospective applications of ultrahigh resolution proteomics in clinical mass spectrometry. Expert. Rev. Proteomics.

[107] Gallien S., Domon B. (2015). Advances in high-resolution quantitative proteomics: implications for clinical applications. Expert. Rev. Proteomics.

[108] Bourmaud A., Gallien S., Domon B. (2016). Parallel reaction monitoring using quadrupole-Orbitrap mass spectrometer: principle and applications. Proteomics.

[109] Nilsson J. (2019). Synthetic standard aided quantification and structural characterization of amyloid-beta glycopeptides enriched from cerebrospinal fluid of Alzheimer's disease patients. Sci. Rep..

[110] Shipman J.T., Nguyen H.T., Desaire H. (2020). So you discovered a potential glycan-based biomarker; now what? We developed a high-throughput method for quantitative clinical glycan biomarker validation. ACS Omega.

[111] Tang K., Page J.S., Smith R.D. (2004). Charge competition and the linear dynamic range of detection in electrospray ionization mass spectrometry. J. Am. Soc. Mass Spectrom..

[112] Annesley T.M. (2003). Ion suppression in mass spectrometry. Clin. Chem..

[113] Chinoy Z.S. (2018). Chemoenzymatic synthesis of asymmetrical multi-antennary N-glycans to dissect glycan-mediated interactions between human sperm and oocytes. Chemistry.

[114] Gagarinov I.A. (2017). Chemoenzymatic approach for the preparation of asymmetric Bi-, Tri-, and tetra-antennary N-glycans from a common precursor. J. Am. Chem. Soc..

[115] Echeverria B. (2015). Chemo-enzymatic synthesis of (13)C labeled complex N-glycans as internal standards for the absolute glycan quantification by mass spectrometry. Anal. Chem..

[116] Ruhaak L.R. (2010). Glycan labeling strategies and their use in identification and quantification. Anal. Bioanal. Chem..

[117] Chen Z. (2017). Development of a hydrophilic interaction liquid chromatography coupled with matrix-assisted laser desorption/ionization-mass spectrometric imaging platform for N-glycan relative quantitation using stable-isotope labeled hydrazide reagents. Anal. Bioanal. Chem..

[118] Gimenez E., Sanz-Nebot V., Rizzi A. (2013). Relative quantitation of glycosylation variants by stable isotope labeling of enzymatically released N-glycans using [12C]/[13C] aniline and ZIC-HILIC-ESI-TOF-MS. Anal. Bioanal. Chem..

[119] Stavenhagen K. (2013). Quantitative mapping of glycoprotein micro-heterogeneity and macro-heterogeneity: an evaluation of mass spectrometry signal strengths using synthetic peptides and glycopeptides. J. Mass Spectrom..

[120] Zhou S. (2016). Reliable LC-MS quantitative glycomics using iGlycoMab stable isotope labeled glycans as internal standards. Electrophoresis.

[121] Li W. (2017). LC-MS/MS determination of a human mAb drug candidate in rat serum using an isotopically labeled universal mAb internal standard. J. Chromatogr. B Analyt. Technol. Biomed. Life Sci..

[122] Panteghini M. (2012). Implementation of standardization in clinical practice: not always an easy task. Clin. Chem. Lab. Med..

[123] Josephs R.D. (2019). Establishment of measurement traceability for peptide and protein quantification through rigorous purity assessment—a review. Metrologia.

[124] Cobbaert C., Smit N., Gillery P. (2018). Metrological traceability and harmonization of medical tests: a quantum leap forward is needed to keep pace with globalization and stringent IVD-regulations in the 21st century!. Clin. Chem. Lab. Med..

[125] De Leoz M.L.A. (2019). NIST interlaboratory study on glycosylation analysis of monoclonal antibodies: comparison of results from diverse analytical methods. Mol. Cell. Proteomics.

[126] Grunwald-Gruber C. (2017). Determination of true ratios of different N-glycan structures in electrospray ionization mass spectrometry. Anal. Bioanal. Chem..

[127] Weykamp C. (2013). Toward standardization of carbohydrate-deficient transferrin (CDT) measurements: III. Performance of native serum and serum spiked with disialotransferrin proves that harmonization of CDT assays is possible. Clin. Chem. Lab. Med..

[128] Helander A. (2017). Reprint of Standardisation and use of the alcohol biomarker carbohydrate-deficient transferrin (CDT). Clin. Chim. Acta.

[129] Cao C. (2020). Absolute quantitation of high abundant Fc-glycopeptides from human serum IgG-1. Anal. Chim. Acta.

[130] Reiding K.R. (2016). Ethyl esterification for MALDI-MS analysis of protein glycosylation. Methods Mol. Biol..

[131] Reiding K.R. (2017). Human plasma N-glycosylation as analyzed by matrix-assisted laser desorption/ionization-fourier transform ion cyclotron resonance-MS associates with markers of inflammation and metabolic health. Mol. Cell. Proteomics.

[132] Galermo A.G. (2019). Development of an extensive linkage library for characterization of carbohydrates. Anal. Chem..

[133] Zhang Z. (2012). Complete monosaccharide analysis by high-performance anion-exchange chromatography with pulsed amperometric detection. Anal. Chem..

[134] Xia Y.-G. (2018). A modified GC-MS analytical procedure for separation and detection of multiple classes of carbohydrates. Molecules.

[135] Xu G. (2017). Revisiting monosaccharide analysis – quantitation of a comprehensive set of monosaccharides using dynamic multiple reaction monitoring. Analyst.

[136] Koles K. (2004). N- and O-glycans of recombinant human C1 inhibitor expressed in the milk of transgenic rabbits. Glycobiology.

[137] Ludwig K.R., Schroll M.M., Hummon A.B. (2018). Comparison of in-solution, FASP, and S-trap based digestion methods for bottom-up proteomic studies. J. Proteome Res..

[138] Wisniewski J.R. (2009). Universal sample preparation method for proteome analysis. Nat. Methods.

[139] Zougman A., Selby P.J., Banks R.E. (2014). Suspension trapping (STrap) sample preparation method for bottom-up proteomics analysis. Proteomics.

[140] van den Broek I. (2013). Evaluation of interspecimen trypsin digestion efficiency prior to multiple reaction monitoring-based absolute protein quantification with native protein calibrators. J. Proteome Res..

[141] Schömig V.J. (2016). An optimized purification process for porcine gastric mucin with preservation of its native functional properties. RSC Adv..

[142] Falck D. (2015). Glycoforms of immunoglobulin g based biopharmaceuticals are differentially cleaved by trypsin due to the glycoform influence on higher-order structure. J. Proteome Res..

[143] Plomp R. (2018). Comparative glycomics of immunoglobulin A and G from saliva and plasma reveals biomarker potential. Front. Immunol..

[144] Horvath A.R. (2014). From biomarkers to medical tests: the changing landscape of test evaluation. Clin. Chim. Acta.

[145] Monaghan P.J. (2016). Biomarker development targeting unmet clinical needs. Clin. Chim. Acta.

[146] Lord S.J. (2019). Setting clinical performance specifications to develop and evaluate biomarkers for clinical use. Ann. Clin. Biochem..

[147] Hilden J. (2016). Early-phase studies of biomarkers: what target sensitivity and specificity values might confer clinical utility?. Clin. Chem..

[148] Considine E.C. (2019). The search for clinically useful biomarkers of complex disease: a data analysis perspective. Metabolites.

[149] Blonder J., Issaq H.J., Veenstra T.D. (2011). Proteomic biomarker discovery: it's more than just mass spectrometry. Electrophoresis.

[150] Hernandez B., Parnell A., Pennington S.R. (2014). Why have so few proteomic biomarkers “survived” validation? (Sample size and independent validation considerations). Proteomics.

[151] Kyselova Z. (2008). Breast cancer diagnosis and prognosis through quantitative measurements of serum glycan profiles. Clin. Chem..

[152] Lee S.B. (2020). Breast cancer diagnosis by analysis of serum N-glycans using MALDI-TOF mass spectroscopy. PLoS One.

[153] Gudelj I. (2015). Estimation of human age using N-glycan profiles from bloodstains. Int. J. Legal Med..

[154] Agre P. (2016). Training the next generation of biomedical investigators in glycosciences. J. Clin. Invest..

[155] Ruhaak L.R. (2013). Chip-based nLC-TOF-MS is a highly stable technology for large-scale high-throughput analyses. Anal. Bioanal. Chem..

[156] Gornik O. (2009). Stability of N-glycan profiles in human plasma. Glycobiology.

[157] Hennig R. (2016). Towards personalized diagnostics via longitudinal study of the human plasma N-glycome. BBA.

[158] Siest G. (2013). The theory of reference values: an unfinished symphony. Clin. Chem. Lab. Med..

[159] Fraser C.G. (2011). Reference change values. Clin. Chem. Lab. Med..

[160] Van Eyk J.E., Sobhani K. (2018). Precision medicine. Circulation.

[161] Bossuyt P.M. (2019). Laboratory measurement's contribution to the replication and application crisis in clinical research. Clin. Chem..

